# The Potential Role of Hedgehog Signaling in the Luminal/Basal Phenotype of Breast Epithelia and in Breast Cancer Invasion and Metastasis

**DOI:** 10.3390/cancers7030866

**Published:** 2015-09-16

**Authors:** Arwa Flemban, David Qualtrough

**Affiliations:** 1 Department of Biological, Biomedical and Analytical Sciences, Faculty of Health and Applied Sciences, University of West of England, Bristol BS16 1QY, UK; 2 Department of Pathology, Faculty of Medicine, Umm Al-Qura University, Makkah 24382, Saudi Arabia; E-Mail: aflemban@uqu.edu.sa

**Keywords:** breast cancer, hedgehog signaling, metastasis, luminal breast cancer, basal-like breast cancer, epithelial mesenchymal transition

## Abstract

The epithelium of the lactiferous ducts in the breast is comprised of luminal epithelial cells and underlying basal myoepithelial cells. The regulation of cell fate and transit of cells between these two cell types remains poorly understood. This relationship becomes of greater importance when studying the subtypes of epithelial breast carcinoma, which are categorized according to their expression of luminal or basal markers. The epithelial mesenchymal transition (EMT) is a pivotal event in tumor invasion. It is important to understand mechanisms that regulate this process, which bears relation to the normal dynamic of epithelial/basal phenotype regulation in the mammary gland. Understanding this process could provide answers for the regulation of EMT in breast cancer, and thereby identify potential targets for therapy. Evidence points towards a role for hedgehog signaling in breast tissue homeostasis and also in mammary neoplasia. This review examines our current understanding of role of the hedgehog-signaling (Hh) pathway in breast epithelial cells both during breast development and homeostasis and to assess the potential misappropriation of Hh signals in breast neoplasia, cancer stem cells and tumor metastasis via EMT.

## 1. Structure and Development of the Breast

The hedgehog pathway functions in controlling cell proliferation, cell fate, and patterning, as well as stem and progenitor cell maintenance, self-renewal and tissue repair [1,2,3,4,5]. This pathway is critical for homeostasis in mature tissue through the maintenance of somatic cell numbers within the organs of the body [3,5].

The human breast contains two types of epithelial cells, referred to as epithelial and myoepithelial, which form the mammary acini. These are linked to the branching ductal structures, forming the tubular-alveolar gland. The tubular structures terminate at the nipple, and function to transport milk produced in the acini. There is also a supporting stromal tissue, that is composed mainly of fat, known as the fat pad, which surrounds these tubular-alveolar structures of the mammary gland [6].

**Figure 1:**
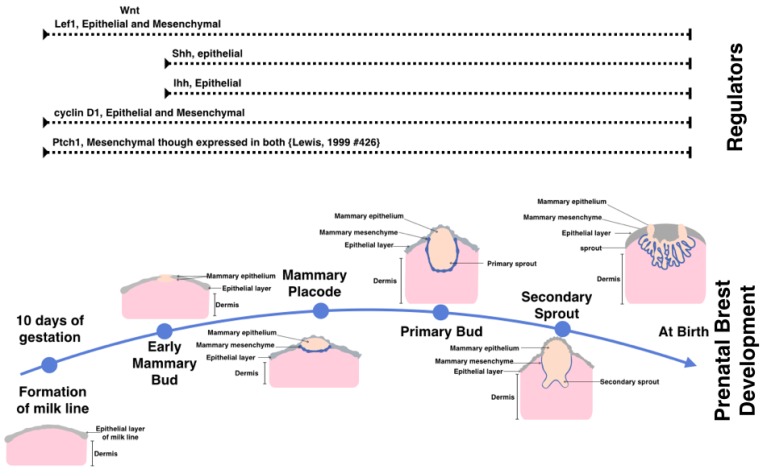
The prenatal stages of mammary gland tissue development. The mammary gland forms as early as day 10 of gestation in mice by the formation of the milk line. This is followed by at least four stages of development. At the time of birth the breast tissue is composed of three distinct layers including the mammary epithelium, mammary mesenchyme. (Which forms the structure of the secondary sprouts), and the dermis. Above are the expression patterns of active signaling and necessary for prenatal mammary gland formation. Illustrated in the top are the hedgehog components associated with those stages of development in mouse embryonic study.

**Figure 2:**
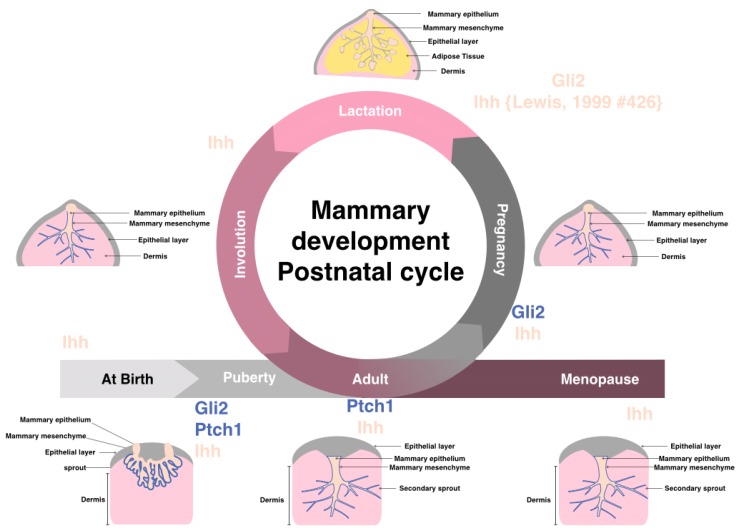
Postnatal mammary gland development is composed of several stages (after birth, puberty, adult, pregnancy, lactation, involution, and menopause). Highlighted are the known key hedgehog signaling components expression found essential at each stage of mammary adult female breast. It should be noted that other Hh components are found expressed at various stages, yet the highlighted compounds are the ones found important for that stage of development.

During embryogenesis mammary tissue arises from the ventral ectoderm, formed as a result of interactions between underlying mesenchymal cells and the epithelial cells of the ectoderm [7]. Breast development has two distinct stages, the prenatal stage is summarized in Figure 1, and the postnatal stage shown in Figure 2. Regulation of the embryonic stage of mammary development has not yet been elucidated in its entirety. It has, however, been shown that hedgehog signaling is vital for the early development of the mammary gland, with both sonic-(*Shh*) and Indian-hedgehog (*Ihh*) expressed and required in mammary epithelium [7].

**Figure 3:**
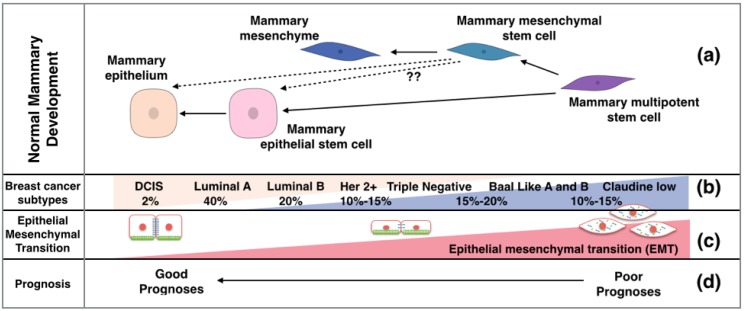
The mammary gland stem cell types and the corresponding breast cancer type. (**a**) The expansion of breast cell types from a single progenitor; (**b**) The molecular sub classification of breast cancers, their hypothesized origin, and percentage prevalence at clinical presentation; (**c**) The involvement of EMT process in breast cancer and a suggested level of EMT according to the sub classification of breast cancer; (**d**) The associated prognosis of various breast cancer subtypes.

The mammary epithelium consists of several lineages; including luminal, alveolar and myoepithelial cells. The normal mammary tissue contains both luminal and mesenchymal (or myoepithelial) stem cells, responsible for renewal of the breast epithelium, as well as the massive expansion associated with cycles of pregnancy [8]. Both luminal and mesenchymal stem cells are needed to form the normal structure of the mammary gland. It has been demonstrated that transplantation of mesenchymal stem cells alone leads to the production of both luminal and mesenchymal cell types (Figure 3), although the mechanism for this is not clear [8].

The development of the mammary gland begins with the formation of a rudimentary ductal tree in early embryogenesis, from the epithelial milk line, which is formed by the thickening of mammary ridge epidermis along the ventral surface, however, the majority of breast development occurs after puberty [4,9]. Hedgehog signaling is vital for the development of many organs in the body, including the mammary gland. Hedgehog genes have an active role during every phase of breast development both pre-and post-natal [5,10]. Figure 2 shows the cycle of postnatal breast development, with genetic analysis in mice indicating a role for hedgehog signaling in mammary ductal morphogenesis, and in human mammary epithelial stem cell self-renewal [4].

The function of Shh in the lactating breast has been reported to be restricted to paracrine, inter-epithelial signaling, and no evidence has shown any autocrine function with the responding cells found in the tissue adjacent to the secreting cells [11]. Furthermore, this paracrine signaling was restricted to the surrounding responsive mature epithelial cells, and not the nearby mesenchymal cells, which were unaffected.

## 2. Breast Cancer Presents Either a Basal or Luminal Phenotype

Breast cancer is the second leading cause of death in women worldwide, with one in eight women developing this disease, and is the most diagnosed cancer in females. It is classified histologically according tissue morphology, into ductal and tubular types, which is further divided into benign: ductal carcinoma *in situ* (DCIS); and lobular carcinoma *in situ* (LCIS), or invasive: invasive ductal carcinoma (IDC); and invasive lobular carcinoma (ILC) [12]. Breast cancer is further classified into luminal A/B, human epidermal growth factor receptor 2 (HER2)-enriched, basal-like (BL), and claudin-low [13]. Basal like breast cancer is classified in breast cancer cell lines into subtypes A (basal) and B (mesenchymal) [14]. Breast cancer is classified according to the expression of prognostic markers, including estrogen receptor (ER), progesterone receptor (PR) and human epidermal growth factor receptor 2 (HER2) (Luminal A, Luminal B that are ER, PR, and HER2 positive), HER2 only positive, BL (expressing basal cytokeratin); triple negative (TN) (negative for all three receptors) [13]. The basal and triple negative subtypes show considerable overlap (i.e. the majority of basal-type tumors are “triple negative” and *vice versa*). The percentage of each subtype presenting clinically as well as their associated prognosis is summarized in Figure 3.

## 3. The Components of Hedgehog Signaling Pathway

**Figure 4:**
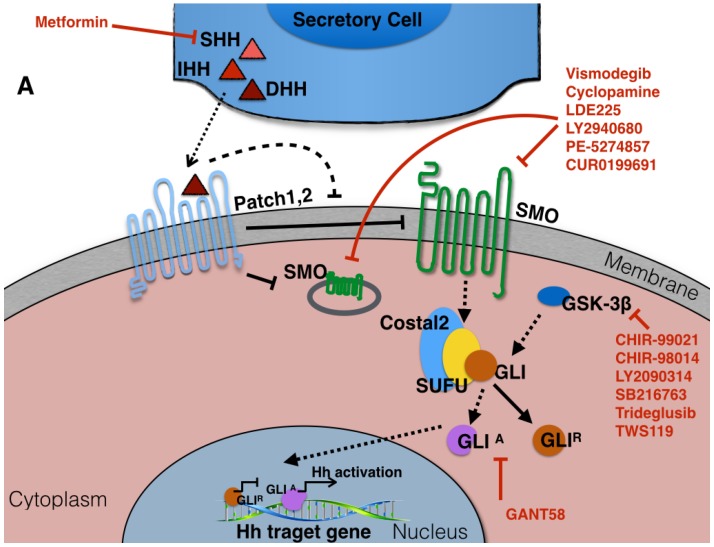

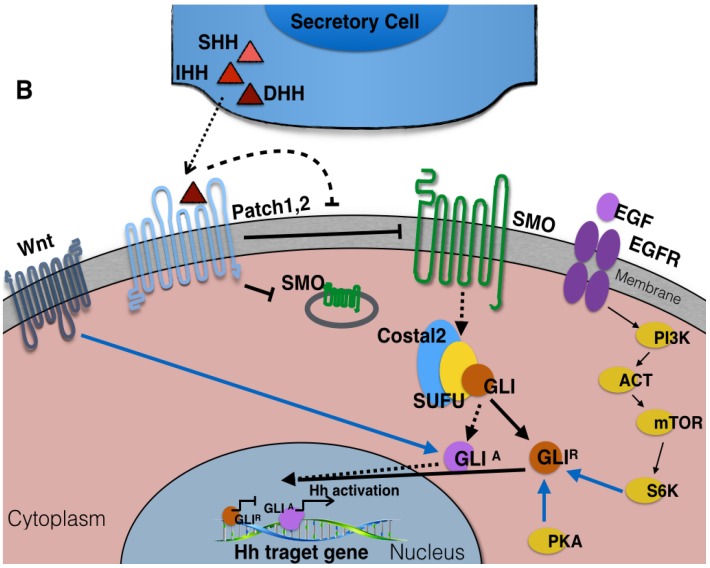
The Hedgehog signaling pathway. (**A**) The canonical hedgehog signaling components, the secreted ligands (Sonic Hedgehog (Shh), Indian Hedgehog (Ihh), and Desert Hedgehog (Dhh)) and the Patched family hedgehog receptors Patched-1 (Ptch-1) and Patched-2 (Ptch-2). Both patched receptors antagonize the function of the Smoothened (Smo) transmembrane effector protein in the absence of the ligand, therefore inhibiting the expression of one or more of the *Gli* (Glioma-associated oncogene homologue) family of transcription factors (Gli-2 or Gli-3) [1,4,5,18]. In the absence of ligand, Gli is sequestered in the cytoplasm by binding to form a large complex protein with the Kinesin-like Costal2 and the serine-threonine kinase Fused [1,2,4,15,18]; (**B**) Hedgehog signaling can be activated through three known non-canonical pathways, including Shh-mediated ERK activation in mammary epithelial cells, Wnt signaling pathway involvement in the expression and function of Gli proteins, and the atypical interaction of core Hh pathway components with each other [15,16,17].

Hedgehog signaling may be transduced by two distinct mechanisms, known as the canonical and non-canonical pathways (both summarized in Figure 4). The canonical pathway (Figure 4A) depends on the interaction between hedgehog ligands from the secreting cells and the Patched (Ptch) receptor on another cell. This interaction then releases the Ptch-mediated inhibition of the Smoothened (Smo) complex, initiating signal transduction in the receiving cell. This results in release of the activated form of Gli (Gli-A in *Drosophila* which correspond to Gli-1 in mammals), which translocates to nucleus, where it acts as transcriptional regulator. It has been shown that both *Gli-1* and *Ptch-1* provide regulatory negative feedback of the cascade [15].

In addition to the canonical Hh signaling pathway, a non-canonical Hh pathway was recently reported [16,17]. This alternate mechanism involves activation of the hedgehog pathway components by other signaling cascades such as that associated with the epidermal growth factor receptor (Figure 4B).

## 4. Hedgehog Signaling in Physiologically Normal Pre- and Post-natal Mammary Gland

Kameda *et al.* demonstrated patterns of Hh signaling during development by studying disruption of their function. Knock-out animal models and transplantation studies in the mouse have shown that hedgehog signaling plays a critical role in ductal development in the mammary gland [2].

Mammary gland tissue arises in embryogenesis as a result of interactions between underlying mesenchymal cells and epithelial cells of the ectoderm, but the regulation of this process in human embryogenesis is not entirely clear [7]. Michno *et al.* showed that hedgehog signaling is vital for mammary gland development in animal models. Both *Shh* and *Ihh* are expressed during breast tissue development, where they are expressed exclusively in the mammary epithelium. Furthermore, when one of these genes was knocked-out the other was able to compensate for its absence [7]. Gritli-Linde *et al.* showed that the mammary gland shares a common progenitor with the hair follicle, both arising from the dermis. Knocking down *Shh* in the earliest hair follicle progenitor tissue results adoption of a mammary gland fate, and knocking down *Ihh* in the early stages does not affect hair follicle development [9]. On the other hand, knocking-down *Shh* does not affect the early development of mammary gland. Thus, suggesting that *Ihh* is the key regulator in early development of epithelial tissue of the mammary gland, whereas absence of *Shh* is apparently important for tissue to follow hair follicle fate. Additionally, *Shh* knockout mice, though die prematurely as Shh is vital for development, has normal uninterrupted breast development in all embryonic stages. One of the earliest known markers for mammary bud formation, *Lef-1*, was unchanged in the absence of *Shh* [7]. The expression of *cyclin D1*, a target of the Hh pathway, in *Shh*-deficient mice was unaltered compared with that of wild type mice [7].

*Ptch-1* is expressed in both the epithelial and mesenchymal tissue of the developing mammary gland and is essential in mammary morphogenesis [7]. Heterozygous *Ptch-1*-deficient mice developed ductal hyperplasia and dysplasia during puberty and virginal adulthood, mainly in ductal structures, with minor defects associated with terminal buds [5,7]. This dysplasia reversed during pregnancy and lobuloalveolar structures matured to facilitate lactation. Lewis *et al.* showed that high expression of *Ihh* during pregnancy and lactation compensated for absence of *Ptch-1* (Figure 2). Then, the tissue exhibited dysplasia after involution and breast remodeling, [5], where *Ptch-1* expression becomes undetectable 2 days after involution. Furthermore, staining of the dysplasia showed that it did not reach myoepithelial tissue, suggesting the function of *Ptch1* is restricted to areas adjacent to the dysplastic epithelial tissue, no detectable expression was seen distant from epithelial cells [5]. Michno *et al.* showed that *Ptch-1* was required in the mesenchymal tissue and not in the epithelial cells, as transplantation of epithelial cells from *Ptch-1* haploinsufficient mice to wild type mice led to formation of normal mammary ducts [4,7]. Also, increased expression of activated human *Smo-2* in transgenic mice leads to increased proliferation, altered patterns of differentiation, and ductal dysplasia that is distinct from the pattern associated with *Ptch-1* heterozygosity [4].

Various studies have shown the importance of *Gli* transcription factors, the downstream effectors of Hh signals, in mammary gland development in the embryo. Gritli-Linde *et al.* showed that transplantation of *Gli-2* knock-down epithelial cells into wild type fat tissue resulted in normal growth of mammary tissue, suggesting that the required Hh signaling arises from the underlying mesenchymal tissue [7]. A recent study reported that Gli-3 is required during the early stages of mammogenesis, and *Gli-1* transcription and *Gli-2* function were antagonized early in development of the breast [15]. The data published by both Lee *et al.* and Michno *et al.* suggested that there was a differential requirement for Gli activity between mammary epithelium and mesenchyme, as they reported that *Gli-2 and Gli-3* were expressed in later stages of embryological development [7,15]. However, the effect of expression of one *Gli* family member on the expression of the others has not been clarified yet.

In the normal mammary tissue of mice, the expression of *Shh*, *Ihh*, and *Dhh* have been identified by RT-PCR, and only *Ihh* expression was detected by *in situ* hybridization, where it was expressed near the terminal buds at the point where the ducts branch [7]. *Patch-1* was expressed in the epithelial cells as well as the fibroblasts in surrounding mesenchymal tissue [7]. Undifferentiated luminal epithelial cells of terminal end buds were expressing higher levels of *Ptch-1* compared with the mesenchymal stem cells. During puberty, the pattern of expression of *Ptch-1* is restricted to the terminal end buds, illustrating its key role in the regulation of mammary ductal tree growth [5]. Gli-2 protein is expressed in the mesenchymal cells near the end buds during puberty, and in early pregnancy, whereas in the later stages of pregnancy it is expressed in the alveolar epithelial cells [7].

Therefore, there is a clear requirement for Hh signaling in the mammary gland. Certain components of the Hh pathway are important in early breast development, and disturbance of this pattern of expression was associated with abnormalities including dysplasia. Thus suggesting a potential role for Hh in the regulation of homeostasis and therefore, potentially, neoplastic transformation.

## 5. Hedgehog-Signaling Pathway in Breast Cancer

Hedgehog signaling is altered in nearly 25% of all human cancers [1,3,4,5]. There is evidence in the literature that the proliferative capacity of various cancer cell lines is reliant, at least in part, on Hh signaling [2,19], and some studies have suggested that Hh signaling plays a key role in tumor cell invasion [2,18]. Dysregulation of the pathway has been linked with the formation and malignant progression of lung, breast, pancreatic and prostate cancer [4,18]. Hh signaling has now also been shown to be associated with the invasion of many cancer cell types including those showing mutational activation of the pathway such as basal cell carcinoma of the skin, and medulloblastoma [2,5]. Recent studies have also linked Hh with invasion in other solid tumors, such as those of the stomach, ovary and lung [2,5,20,21,22].

Hedgehog signaling has been demonstrated to contribute to early breast cancer development by increasing tumor cell proliferation [4], although prior to 1999 there were no published studies on the presence or role of Hh signaling in breast cancer. *Ptch-1* mutations were identified in a small proportion of breast cancers at that time [5]. Zhang *et al.* reported evidence that dysregulation in hedgehog signaling may contribute to breast cancer development in an animal model [1]. In 2009 it was reported that activation of the Hh pathway is a common feature in breast carcinoma, a finding also confirmed in later studies [2,3]. Mutations in *Ptch-1* and *Gli-2* genes in mouse models resulted in abnormalities in mammary ductal morphogenesis, including hyperplasia that resembled hyperplasia in humans [2]. In the initial studies, little or no evidence of a connection between mutations in the Hh signaling pathway and breast cancer was reported, with no mutations in either *Shh*, *Ptch-1*, or *Smo* [23,24], confirming the earlier work showing that mutational Hh pathway activation was uncommon in these tumors. Table 1 shows a summary of the published reports investigating Hh pathway mutations in breast cancer, with rare somatic mutations of *Gli-3* also published [15]. It should be emphasized here that even though Hh pathway mutations are rare in breast cancer, similar to many of the other common human malignancies, it has been demonstrated that this pathway is important the pathogenesis of many human cancers including colon cancer [19].

Mutations are not the only mechanism by which Hh signaling can promote cancer. Misappropriation of Hh signaling, that is increased or decreased pathway activation is frequently seen in human cancers, such as colon cancer [19]. Therefore, for the purpose of this review, this group of signaling defects will be further discussed. Localization of some of the Hh signaling components, Gli in particular, is important to determine the functional status of the pathway. For example, localization of Gli in the nucleus indicates functionally active Hh signaling (Table 2).

**Table 1:** Mutations in hedgehog signaling pathway associated with breast cancer.

Type of Mutation	Study	Genes	Finding in Breast Cancer Studies	Ref.
Missense mutation	Mice	*SHH*	1/6 mice developed cancer	[25]
Missense mutation	Human DNA compared to normal tissue	*Ptch-1*	2/7 breast carcinoma	[26]
Missense mutations	Human breast cancer cell lines and primary breast tumors	*Gli-1*	2/24, 8% breast cancers 9% breast cancer cell lines	[27]
Loss of chromosomal region	Human breast cancer cell lines and primary breast tumors	*Ptch-1*	19% of primary breast cancers 33% breast cancer cell lines	[28]
Polymorphism	Human clinical samples	*Ptch-1*		[29]

**Table 2:** Expression of hedgehog in breast cancer.

Hh Component	Expression in Breast Cancer Compared to Normal	Study and Reference
Gli-1 *mRNA* and protein	40%–100% Increase	Cell lines and Human clinical samples [30]
		Cell lines [31]
		Cell lines compared to human mammary epithelial cells (HMEC) [32]
		Human tissue from primary tumors and metastasis site [33]
		Human tissue from primary tumors [34]
		Breast cancer cell line [35]
		Human clinical samples [36]
Ptch-1 *mRNA* and protein	40% Decrease; 50%–58% Decreased protein or 33%–96%increase	Cell lines [31]
		Breast cancer cell lines [37]
		Human clinical samples and transgenic mice [4]
		Breast cancer cell lines and clinical samples [38]
		Clinical samples and cell lines [30]
		Cell lines and clinical samples [31]
		Human clinical samples [36]
		Human sample from primary tumors [34]
		Breast cancer cell lines [35]
Smo *mRNA* and protein	30%–70% Increase	Transgenic mice and clinical samples [4]
		Cell lines and clinical tissue [31]
		Human samples from primary tumor [34]
		Breast cancer cell lines [35]
		Human clinical samples [36]
Shh Protein and *mRNA*	63%–100% Increase	Cell lines and Human clinical samples [30]
		Cell lines and clinical tissue [31]
		Human samples from primary tumor [34]
		Breast cancer cell lines [35]
		Human clinical samples [36]

In 2008, alternative splice isoform of Gli1 was identified (GLIΔN), with wild type GLI1 full-length (GLI1FL) expression was more abundant in the nucleus than this isoform [39]. The activation capacity of GLI1ΔN was reported to be more than that of GLI1FL though the latter is found more concentrated in the nucleus. Later in 2009, another splice variant was identified and was called truncated GLI1 (tGLI1) [40]. A growing body of evidence suggests that there is variation in expression pattern of these different isoforms, and that there is a difference in their ability to regulate gene activation [40,41]. These differences lead to changes in cellular behavior including proliferation, migration, invasion, angiogenesis, and metastasis. The structure and regulation of the GLI1 isoforms by Hh signaling pathways have been reviewed previously [42,43]. tGLI1 was reported to be frequently expressed in human breast cancer cell lines and primary breast cancer specimens, but was not detectable in normal breast tissue [41]. It has been shown to upregulate vascular endothelial growth factor-A (VEGF-A), thereby stimulating angiogenesis. Also, tGLI1 enhanced the ability of breast cancer cells to migrate and invade, by facilitating anchorage-independent growth [41]. These findings are supported by a later report showing increased expression of tGLI1 supporting tumor angiogenesis and aggressive growth in glioblastoma [44].

Various reports show that this type of inappropriate activation of Hh signaling also occurs in breast cancer. The *Ptch* promoter is methylated in some breast cancer cell lines, and this correlates with low *Ptch* expression [38]. *Gli-1* upregulation correlates with poor prognosis of triple negative and basal like breast cancers. Limited *Gli-3* mutations are limited and not reported in cancer samples [15] and over expression of *Gli-1* is associated with poor prognosis [45].

There is debate in associating Hh deregulation within the various subtypes of breast cancer. Hh signaling was shown significantly higher in invasive ductal carcinoma compared with non-invasive ductal carcinomas (DCIS). In 2009, a group of researchers in Japan showed that Hh is constitutively activated in surgically resected ER-receptor negative breast cancers [2]. Nuclear localization of Gli-1 was detected in both ER-positive as well as ER-negative cases, and suggested the exploitation of *Gli-1* as target for therapy [2]. Quantitative analysis using Real-Time RT-PCR on clinical samples, containing 4 Triple Negative and Basal-Like breast cancer as well as six HER2 positive cases, showed higher *Gli-1* expression in tumors compared with matched normal tissue. With ER-receptor status being significantly associated with Hh activity. Whereas *Gli-1* and *Ptch-1* mRNA and protein are expressed in triple negative breast cancer cell lines [2]. Knocking down Gli-1 in in two ER receptor negative cell lines, one triple negative and one basal resulted in significantly reduced proliferation in both cell lines [2]. Knock down of *Gli-1* or inhibition of *Smo*, by the plant-derived inhibitor cyclopamine, resulted in decreased invasive capability in these cell lines. Table 2 summarizes the published reports of Hh pathway expression in breast tumors However, it is worthy of note that differences in expression of Hh signaling between the different molecular classifications of breast cancer was not factored into most of these studies.

Zhang *et al.* recently showed that inhibition of Hh signaling with cyclopamine resulted in decreased growth of breast cancer cells *in vitro* [1]. The same study showed altered expression of hedgehog network genes in clinical samples and breast cancer cell lines, showing active Smo-mediated signaling. It has also been reported that it was not only Gli-1 that was important for breast cancer prognosis but also that there was increased expression of Shh [45] which has been shown to contribute to tumor cell survival [31]. Other studies have shown decreased, or even loss of, Ptch-1 expression in almost 50% of breast cancer cases—with increased Smo detected in approximately 70% of ductal carcinoma *in situ* (DCIS) and 30% of invasive breast carcinoma [1,4]. Expression of *Shh*, *Ptch-1*, *Gli-1*, and *Smo mRNA* in breast cancer tissue was also shown to correlate with disease recurrence.

With the exception of *Gli-1*, all other pathway members were significantly correlated with the size of the tumor, and *Smo* was associated with lymph node involvement [34,46]. Thus, these studies related signs of tumor aggression with elevated levels of Hh pathway expression. These findings have been corroborated by other work [30,47], although contradictory data has also been reported by another group [48]. This apparent controversy can be explained by the different approaches used in the selection of cases and in the detection methods employed.

Recent *in vitro* cell line studies provide more insight into the association of Hh signaling with breast cancer. *Gli-1 mRNA* level was increased in a number of breast cancer cell lines, including MDA-MB-453 (TN), MDA-MB-231 (TN and basal type B), BT20 (basal type A), MCF10A (benign breast cancer cell line) and SKBR3 (HER2^+^) in comparison to a primary human mammary epithelial cells (HMEC) [45]. Gli1 protein expression was significantly higher in two TN and basal cell type B cell lines compared with HMECs. *Gli-1 siRNA* treatment of these cells resulted in decreased cell proliferation with a concomitant increase in apoptosis, and *Gli-1* knock down also resulted in a significant reduction in the migratory ability of these cells [32]. These data suggest an important role for Hh signaling in maintaining tumor cell survival in breast cancer.

*In vivo*, Gli-1 overexpression correlates with unfavorable overall survival in patients, tumor stage, and lymph node involvement [45]. Furthermore, activation of Gli-1 was independent of Hh-ligands and does not depend on Smo either, which signposts a potential alternative pathway of activation (Figure 4B) [32]. A previous study showed that *Shh* genes predicted poor clinical outcome in inflammatory breast cancer patients using microarray analysis [49]. Cell lines derived from inflammatory breast cancers have activated EGFR that similarly points toward alternative activation of Hh through the non-canonical pathway (Figure 4B). These data suggest that Hh could also play a key role in the progression of breast cancer, and may be useful prognostic indicator.

Gli-1 is also expressed in normal breast tissue, suggesting that Gli-1 is involved in regulation of normal breast cell behavior [45,50]. This result remains a debated topic and some studies reported no Gli-1 expression at all in normal breast epithelium [30]. This could be due to variations in tissue sampling, or in the threshold of detection for the protocol used. It may prove beneficial to include comparison of the molecular subtypes of breast cancer in future studies, and this approach could answer the debate about involvement of Gli-1. To this end, a recent study showed that nuclear localization of Gli-1 was associated with the hormone receptor negative, basal-like breast cancer group [50]. Also, it was reported in this study that there was nuclear and cytoplasmic localization of Gli-1 in a small percentage of basal epithelial cells of normal breast tissue. Thus, supporting notion that expression of Gli-1 is expressed in normal breast tissue and that its location in a small percentage of particular cell type and explaining the current question mark over its expression in normal tissue.

Further evidence for differing Hh patterns within the different breast cancer subgroups was illustrated by the finding of Gli-1 and Smo overexpression in TN breast cancer cases and a positive correlation with increased tumor stage. This was offset by an inverse correlation with estrogen receptor (ER) expression, again suggesting subtype-specific expression patterns [36]. *In vitro* studies of Gli-1 have shown high levels of expression in SUM145 cells, which are basal type B as well as triple negative, yet it was not increased in other triple negative and basal-like breast cancer lines, including MDA-MB-231. Thus, suggesting that other factors contribute to high Gli-1 expression in these cells [32]. On the other hand Gli-1 expression was lower in the ER-positive breast cancer cell lines T47D and MCF7 [36]. Studies of *Gli-1 in vivo* report increased expression in four out of five paired normal and tumor clinical samples, which was not statistically significant, which could be due to small sample size [45]. They, and others, have observed increased nuclear localization of Gli-1 in breast cancer samples compared to normal breast tissue, yet there was agreement in two studies that strength of expression of Gli-1 varied significantly among breast cancer samples [30,45].

The localization of *Gli-1*, nevertheless, has raised a debate over its overexpression and the presence or absence of hormone receptors in breast cancer [30,45,50], with some studies reporting a correlation between the nuclear localization of Gli-1 and ER status [30], some linking Shh expression and ER-alpha in particular [51,52]. Others suggest that this pathway presents a potential target in ER-negative carcinomas [2]. Jeng *et al.* in 2014 reported that they did not find a correlation between Shh and ER or progesterone receptor (PR), but it was related to Her-2 overexpression [34]. Another group reported a correlation between ER and Ptch expression and that Ptch polymorphisms are linked to cancer risk associated with oral contraceptive use [38].

Ptch-1 was shown to be increased significantly in luminal breast cancer cell lines MCF7, T47D, 13762 and HER2+ cell line SKBR3 [37]. *Ptch-*1, on the other hand, was reported to regulate cell cycle progression and high expression of *Ptch-1* has been associated with metastasis in many human cancers [53]. It was observed that expression of Ptch-1 was significantly decreased in breast cancer compared to mammary hyperplasia [36]. A study suggested that the *mRNA* of *Ptch-1 and Gli-1* in core needle biopsy would provide a useful tool for surgeons and patients for the selection of treatment options in the clinical management of breast cancer [34].

Recently, a study reported that increased expression of a liver kinase B1 (LKB1) in breast cancer cell lines inhibits Hh signaling, as well as decreasing the rate of growth of the cell lines in a xenograft model. This study determined that LKB1 controls cellular proliferation and induces programmed cell death. Conversely, LKB1 knock down in the same cells resulted in significant activation of the Hh pathway and a significant increase in xenograft growth of these cells when injected into mice [18]. LKB1 antagonized the function of Hh protein expression in MDA-MB-231 cells and cells with active Hh signaling have decreased or non-detectable LKB1, therefore, suggesting that LKB1 is a negative regulator for Hh [18]. Staining of histological clinical specimens of ductal carcinoma cases revealed that there is high expression of LKB1 in 44% of cases and that the expression of this protein negatively correlates with Shh, Gli-1, and Smo, while there was no observed correlation with Ptch [18]. There appears to be some contradiction amongst the reported levels of expression of Ptch-1, as mentioned previously. This apparent variation in findings may be due to different locations from which biopsies were selected, as well as variation in the method of investigation.

Target genes for Gli transcription factors include *Ccnd 1*, *Bcl-2* and members of the *Myc* gene family, which affect cell cycle, survival, proliferation, stem cell activation, as well as metastasis [54]. High levels of *Ccnd1* were reported in GLI1-induced highly proliferative mammary gland lesions [55]. Another target gene called *BCL-2* is commonly found up-regulated in human tumors including breast cancer [56,57]. Also, the transcription factor Snail is elevated after ectopic expression of GLI1 in the mouse mammary gland, which leads to loss of E-cadherin expression, thus promoting EMT, discussed later [58].

Taken together, these data suggest an important role for Hh signaling in breast cancer development and progression. What is not yet clear, however, is how Hh activation relates to the specific tumor subtypes, and therefore how this could impact on prognosis. A key issue affecting patient survival is disease recurrence. In recent years we have come to understand the importance of cancer stem cells (CSCs) in the ability of a tumor to return following treatment. Although the study of CSCs has yielded a wealth of data and understanding, many questions remain unanswered. Hh signaling is known to modulate the stem cell phenotype in a number of tissues and, as described above, has been implicated in stem cell maintenance in healthy breast tissue. The role of Hh in breast CSCs is not yet clear.

## 6. Breast Cancer Stem Cells

A recent study reported that normal mammary tissue has two distinct types of stem cells (see above) [8]. The molecular sub-classification of breast cancer identified five major subtypes of breast tumors, as indicated previously, and each of these sub-classifications have significantly different prognosis and treatment strategies and, as each presents with a different cellular phenotype, it is possible that they potentially arise from different progenitor cells [59].

To facilitate studying the behavior of mammary stem cells it is necessary to have available an *in vitro* model. Human mammary epithelial cells (HMECs) have been successfully cultured and provide a model to study mammary stem cells, retaining the ability to undergo the stem cell-like epithelial mesenchymal transition [59].

It has been hypothesized that continuous activation of Shh may neoplastically transform breast stem cells and be associated with a poor cancer prognosis [36]. A later study suggested that the activation of Shh pathway was not only vital in early stages of tumorigenesis by initiating aberrant stem cell growth, but is also important for subsequent cancer progression and recurrence [34]. Moreover, it was reported that there were luminal progenitor cells in a population of luminal epithelial cells that are Hh-sensitive in the breast as well as basal progenitor cells in that population [11]. Hh signaling is important for progenitor cell proliferation and differentiation [60] and misappropriation of the Hh signaling pathway in this population of cells may lead to increased progenitor growth and subsequently lead to cancer [11].

High levels of SMO and Gli-1 expression have been found to correlate with continuous activation of breast cancer stem cells in TNBC patients samples [36]. Furthermore, another recent study reported that inhibition of NFκB significantly reduced *Gli-1* expression and protein levels in breast cancer cell lines BT549, HS578T, MDA-MB-231, MDA-MB-157, MDA-MB-436, as well as MCF10A [61], and suggested that *Gli-1* functions in the maintenance and regulation of cancer stem cells.

## 7. Hedgehog in the Regulation of EMT in Breast Cancer

It is now widely accepted that the epithelial-mesenchymal transition (EMT) plays an essential role in the metastatic spread of many tumor types, including breast cancer. During localized invasion and subsequent metastasis, cancer cells lose their epithelial characteristics including cell-cell adhesion and polarity markers, and gain mesenchymal characteristics that allow them to become motile and invade neighboring tissue [62,63]. EMT has been linked to the progression of many human epithelial malignancies including: pancreatic [64], lung [65], cervical [66], and colon cancer [67].

Shh up-regulation in tumors is thought to impact on the tumor microenvironment [34] resulting in the formation of activated stroma, which responds to tumor-derived Hh ligands [11,68]. This is different from normal breast tissue in which the nearby mesenchymal cells do not respond to Hh signals originating from active epithelial cells (described above).

It has been shown that the Wnt signaling pathway is involved in the regulation of breast progenitor cell proliferation. Hh and Wnt classically act together, and reciprocally, to regulate cell behavior across epithelial-mesenchymal boundaries in both the developing embryo and in adult tissues. Recent work has shown that misappropriation of both Wnt and Hh signaling between epithelial progenitors and adjacent mesenchymal cells leads to mammary tissue hyperplasia in a mouse model [11]. Hh and Wnt have also been shown to be important mediators of EMT, both in the developing embryo and in tumor progression. Key features of EMT include the loss of expression of the cell-cell adhesion molecule E-cadherin and expression of transcription factors such as Slug and Snail. In SUM145 cells (basal type B), it has been shown that Gli-1 inhibits E-cadherin expression. However, Gli-1 did not affect the levels of the transcription factor Snail, a known repressor of E-cadherin, suggesting an alternate mechanism [32]. The authors suggest that further investigation of the mechanism of Gli-1-mediated E-cadherin regulation, and the relationship to EMT, is required [32].

## 8. Potential Therapeutic Targeting of the Hedgehog-Signaling Pathway

As our understanding of the role of Hh in human disease, particularly cancers has increased, there has been great interest in targeting this pathway therapeutically. There are several naturally-occurring substances with an ability to antagonize hedgehog signaling, and a number of hedgehog antagonists have been synthesized (Figure 4). The widely used hedgehog inhibitor cyclopamine belongs to a group of plant-derived alkaloids, first identified for their teratogenic activity in sheep [1]. Cyclopamine acts through direct binding to Smo, therefore, inhibiting the downstream canonical Hh signaling pathway [69]. It has also been shown to inhibit the non-canonical Akt pathway, although the precise mechanism for this is not clear [3,70]. *In vitro* studies in other tumor types have also shown promise, such as the demonstration of cyclopamine-induced apoptosis in colon cancer cells [19].

Recently, a study reported that the diabetes drug metformin lowered the risk of certain types of cancers, by inhibiting the Shh signaling pathway [35]. Furthermore, this drug inhibited breast cancer cell migration and invasion as well as cancer stem cell survival and self-renewal, accompanied by a dose dependent decrease in the levels of several Hh components. Studies in mice of with other hedgehog inhibitors showed minor or non-significant side effects, thus, highlighting their potential use in treatment of cancer [70].

Cyclopamine has been shown to inhibit cellular proliferation in a dose-dependent manner in the breast cancer cell line MDA-MB-231 (triple negative, basal type B) [2]. Others have investigated the use of cyclopamine in combination with paclitaxel, commonly used in breast cancer chemotherapy. They reported that this combination resulted in increased tumor cell line apoptosis compared to paclitaxel treatment alone [71]. Furthermore, this study also showed that blocking hedgehog resulted in loss of tumor stem cell maintenance and led to reduction of cancer stem cell-related chemotherapy resistance. Another study in the same year reported that cyclopamine works as a novel, potent inhibitor of the proliferation of breast cancer cell lines and reduced expression of ER in ER-positive cells [3]. Both MCF7 and MDA-MB-231 cell lines (ER-positive and ER-negative, respectively), displayed significant reduction in growth following cyclopamine treatment [3]. Cyclopamine arrested the cells in the G1 phase of the cell cycle by interrupting cyclin D1 production through modulation of the MAPK/ERK signaling pathway. Cyclopamine also reduced the invasive ability of these cells by inhibiting the expression of NK-κB, MMP2 and MMP9 proteins [3]. Taken together, these data suggest that cyclopamine could be a promising therapeutic agent for the treatment of all subtypes of breast cancer [3].

However, further consideration is needed for validating the usefulness of cyclopamine in treating breast cancer, including dosage and duration of treatment. There is inconsistency in the published reports on the dosage cyclopamine used. Several studies used relatively high doses of the drug for treating cell lines, whilst others used much lower concentrations for treating the same cell lines. No real consensus or justification has yet emerged on the selection of a specific dose [2,3,37].

Similarly, research on the use of cyclopamine *in vivo* has further indicated that its application could be far from straightforward. Nowacka-Zawisza and Krajewska [72] reviewed the benefit of using hedgehog inhibitors alone, or in combination with other agents in the treatment of triple negative breast cancer. However, it is difficult to reach a high systemic levels of cyclopamine *in vivo* because it is potentially toxic and has a relatively short half-life [73]. Conversely, Chai *et al.* reported that they did not observe any effects of cyclopamine, even after 6 weeks of treatment, by histological examination of vital organs [71].

Recently, Colavito *et al.* reported that the issue of drug resistance needs to be factored-in when studying the effect of cyclopamine on breast cancer cell lines, either by elimination of drug from cells or activating Hh signaling through alternative pathway (Figure 4B) [61]. Therefore, further complicating the issue of finding an effective dose of cyclopamine for breast cancer *in vitro* as well as *in vivo* [74]. The other factor to consider is the duration of the treatment. Kameda *et al.* reported that cyclopamine suppressed proliferation of cells after 72 h of incubation, although this effect was not detected after 24 h of incubation [2]. This is supported by data from a recent study that showed that the effect of cyclopamine treatment on breast cancer cell lines started to show significant reduction after 5 to 10 days of incubation [3].

Zhang *et al*. have demonstrated that cyclopamine used to inhibit Smo signaling must be tested in resistant cell lines prior to sensitive ones, in order to eradicate any off-target effects [1]. Furthermore, treatment with cyclopamine or CUR0199691 (another Smo-antagonist) of Smo-expressing and Smo-non-expressing cell lines showed that both agents were able to reduce tumor cell growth at high dosage, regardless of Smo expression status, thus suggesting the presence of secondary target molecule in breast cancer cell lines. This was confirmed by add-back of exogenous Shh, which did not antagonize the effect of cyclopamine in those experiments [1]. As mentioned earlier, off-targets effects may occur at higher doses of cyclopamine treatment—thus the reported effects in studies that treated cells with high doses may not reflect specific effects on hedgehog signaling.

A selective oral inhibitor of SMO called LDE225 has shown an acceptable safety profile after the phase I clinical trials on advanced solid tumors (basal cell carcinoma and relapsed medulloblastoma) [75]. Both responsive cancer types are known to be significantly associated with Hh pathway activation [75]. LDE225 showed greater efficacy than cyclopamine in the *in vitro* reduction of cell proliferation and tumor growth arrest in melanoma [76]. In clinical trials, LDE225 produced a response in medulloblastoma and basal cell carcinoma, as well as disease stabilization in lung adenocarcinoma, spindle cell sarcoma, breast cancer and one case of basal cell carcinoma in a dose-escalation study [75]. LDE225 in combination with Docetaxel is currently in a phase I clinical trial in patients with triple negative advanced breast cancer. The primary objective of this study is to determine the maximum tolerated dose as well as the recommended phase II dose. In this study they will assess the effectiveness of potential biomarkers by evaluating the activation of Hh signaling, the expression of Smo-related biomarkers and Hh target genes, as well as other, related, pathways [77]. However, no published work was found on the effect of LDE225 treatment on breast cancer *in vitro* or *in vivo* at the time of submitting this review.

## 9. Conclusions

Breast cancer metastasis is the cause of death of almost all women diagnosed with this disease. Even though the mortality rate has declined due the introduction of screening and targeted therapy, the survival rates of some breast cancer subtypes remain low. Triple negative and basal-like breast cancer have the worst prognosis, a low survival rate and increased chance of relapse [78]. Therefore, understanding the process of cancer cell metastasis is vital to allow the development of targeted therapy.

The epithelial mesenchymal transition has been associated with human cancer metastasis in a number of tissues, including breast cancer. Therefore, understanding the regulation of this process is important, and Hh presents itself as a potential target. Despite some excellent published work, the role of the Hh pathway in breast cancer remains to be fully elucidated. Many questions still need answering, such as the dosage and treatment duration of the antagonizing agents. The molecular classification of the subtypes of breast cancer in human clinical samples as well as in breast cancer cell lines needs taking into account in studies of this pathway, as they have the potential to utilize Hh to their advantage in different ways and may show different levels of reliance on Hh signals for tumorgenesis and progression. Furthermore, as our understanding of stem cells, both in the normal tissue, and the various subtypes of tumor develop, Hh may again appear as a prime target for therapeutic intervention in at least a subset of breast cancers.

There are apparent contradictions in some of the published work in this area, as there have been in investigations of this pathway in multiple systems. However, an in-depth look at the detail of the published work shows a clear variation between the various subtypes and further emphasizes the need for a subtype-specific approach.

As mentioned previously, Shh, and its ligands, expression are correlated with ER-alpha breast cancers, while Smo and Gli-1 are overexpressed in TNBC. Some studies correlated overexpression of Gli-1 to ER-positive and TN subtypes of breast cancer. Though these data altogether may seem to contradict one another, it would seem that the involvement of Hh signaling pathway in the pathogenesis of breast cancer is mainly caused by misappropriation of these signals, rather than overexpression of a single component of the pathway. Thus, the understanding of this variation in the expression and location of Hh pathway component expression are critical prior to drawing conclusions about potential therapeutic targeting of Hh in a specific group of breast cancer.

There remains a gap in our current understanding of the involvement of Hh signaling in breast cancer, TN and BL in particular, even though there are many studies reporting the involvement of this pathway as key regulator in normal prenatal and postnatal breast development. Hopefully, further study of the association between the Hh pathway and breast cancer will yield new prognostic markers and yield new targeted therapies.
